# Plausibility of Early Life in a Relatively Wide Temperature Range: Clues from Simulated Metabolic Network Expansion

**DOI:** 10.3390/life11080738

**Published:** 2021-07-24

**Authors:** Xin-Yi Chu, Si-Ming Chen, Ke-Wei Zhao, Tian Tian, Jun Gao, Hong-Yu Zhang

**Affiliations:** Hubei Key Laboratory of Agricultural Bioinformatics, College of Informatics, Huazhong Agricultural University, Wuhan 430070, China; chuxy@webmail.hzau.edu.cn (X.-Y.C.); csm930@webmail.hzau.edu.cn (S.-M.C.); zkw000320@webmail.hzau.edu.cn (K.-W.Z.); tiantian@whu.edu.cn (T.T.); gaojun@mail.hzau.edu.cn (J.G.)

**Keywords:** origin of life, metabolism, network expansion simulation, temperature, thermodynamics

## Abstract

The debate on the temperature of the environment where life originated is still inconclusive. Metabolic reactions constitute the basis of life, and may be a window to the world where early life was born. Temperature is an important parameter of reaction thermodynamics, which determines whether metabolic reactions can proceed. In this study, the scale of the prebiotic metabolic network at different temperatures was examined by a thermodynamically constrained network expansion simulation. It was found that temperature has limited influence on the scale of the simulated metabolic networks, implying that early life may have occurred in a relatively wide temperature range.

## 1. Introduction

The temperature of the environment where life originated has elicited a long-term debate. Previous genome sequence-based studies on this issue reached inconsistent results (detailed in the Discussion) [1,2,3,4,5,6,7]. New perspectives are needed to address this issue. Metabolism-first world theory suggests that the origin of metabolism was earlier than the appearance of the genetic system and organic catalysts such as enzymes [8,9,10,11]. The feasibility (thermodynamics) and rates (kinetics) of the reactions that make up metabolism networks are associated with temperature. Therefore, studying the effects of temperature on metabolic reactions and networks may shed more light on the environmental conditions for the origin of life.

The network expansion algorithm has been used to trace the evolutionary history hidden in modern metabolism networks [12,13,14,15]. Using this method, Goldford et al. extracted a phosphorus-independent subset from the full KEGG metabolism, which represented a biosphere-level primitive metabolic network [13]. This network exhibited ancient features, such as the enzymes and protein folds belonging to the speculative last universal common ancestor (LUCA) proteomes, and thus was considered as a “metabolic fossil”. They also showed that thermodynamics is an important limiting factor for the scale of the metabolic network. Interestingly, Goldford et al. found that in standard conditions, a thioester pantetheine instead of phosphates (pyrophosphate or acetyl-phosphate) can significantly alleviate the thermodynamic bottleneck of the expansion of the metabolic network, though the latter is a more common energy source in modern metabolism. Goldford et al. suggested that the phosphorus-independent network provides a solution to the “phosphorus problem”, which was caused by the low solubility and reactivity of Earth’s main phosphate source—apatite [16]. This network also supports the previously proposed thioester world theory, which proposed that in the very early stage of life’s origin, thioesters played multiple important roles in prebiotic metabolism [17]. Recently, based on their phosphorus-independent network, Goldford et al. investigated the impact of several environmental factors on metabolism and found that temperature has a relatively limited influence on the scale of the metabolism network [14].

Nevertheless, phosphorus is an essential element of life and several possible prebiotic phosphorus sources have been proposed [18]. Phosphite, which is more soluble and has higher reactivity than apatite [16], can be generated from volcanic activity [19] or extraterrestrial schreibersite [20]. Another effective phosphorylating agent, polyphosphate, can also be generated in volcanic regions [21]. Phosphite and polyphosphate can be directly used in the synthesis of biomolecules [20,21], or be first converted to orthophosphate in plausible prebiotic environments [22]. Based on these findings, in a recent study [15], we constructed a phosphate-dependent network using Goldford et al.’s method. The obtained phosphate-dependent network could be as ancient as the phosphorus-independent counterpart and could synthesize ribose, which implies a connection between the metabolism world and the RNA world. Moreover, several phosphorylated intermediates such as glucose 6-phosphate can play the same role as thioester, significantly alleviating the thermodynamic bottleneck of network expansion. It is thus intriguing to explore how temperature affects the scale of this simulated phosphorus-dependent network.

## 2. Materials and Methods

### 2.1. Data Sources

The metabolism reactions and compounds were downloaded from the KEGG reaction database (release: 84.0) [23]. The Gibbs free energy of reactions were calculated by eQuilibrator [24,25].

### 2.2. Thermodynamically Constrained Network Expansion Simulation

The thermodynamically constrained network expansion simulation method has been described in detail in previous studies [13,15]. In brief, firstly, all KEGG metabolic reactions were downloaded and vague and unbalanced reactions were filtered out. The remaining reactions and their compounds constituted the background metabolism pool, which included 7376 reactions and 6460 compounds (Table S1). Secondly, the network expansion was started with a seed set of compounds that are considered to have existed on the primitive Earth. The seeds used in the phosphate-dependent network expansion were the same as in our previous study [15], which included water, dinitrogen, carbon dioxide, hydrogen sulfide, ammonia, acetate, formate, and glucose 6-phosphate. The first eight compounds are considered to have been abundant on prebiotic Earth and should participate in primitive biochemical reactions [13,14,15]. Glucose 6-phosphate is a phosphorylated intermediate from glycolysis which was speculated to be prebiotically synthesized [26,27]. Our previous study showed that glucose 6-phosphate can significantly alleviate the thermodynamic bottleneck. Without this compound, the network obtained by thermodynamically constrained expansion contained only dozens of metabolites [15]. In the thioester-dependent network expansion, glucose 6-phosphate was replaced by pantetheine. Note that Goldford et al. proposed that pantetheine can function as CoA thioesters [13]. Therefore, CoA and acetyl-CoA were also added into the seed set. Thirdly, the products of thermodynamically reachable reactions from the background metabolism pool enabled by available substrates were iteratively added into the compounds set until no new reactions or compounds could be produced.

Previously, the thermodynamically accessible standard of a reaction at 25 °C was defined as Gibbs free energy below 30 kJ/mol [13,15]. However, this standard is not appropriate at other temperatures. In this work, we calculated the lowest reaction free energy at the boundary of the physiological range of metabolite concentrations, referring to Goldford et al.’s recent study [14]. Briefly, the free energy of every reaction at different temperatures ($\Delta G^{\prime }$) was calculated using the following equation:

(1) $$\Delta G^{\prime }=\Delta G^{{}^{\circ}\prime }+RTln\prod_{i}a_{i}^{s_{i}}$$

where $\Delta G^{{}^{\circ}\prime }$ is the Gibbs free energy under standard molar conditions, which was estimated by the eQuilibrator program using a group contribution method [24,25]. $\Delta G^{{}^{\circ}\prime }$ is affected by pH, ionic strength, and also by the concentration of free Mg^2+^ (pMg) for some reactions like ATP hydrolysis [25]. These conditions were set as follows: pH = 7.0, ionic strength = 0.1 M, and pMg = 0.0, which is the same as our previous study [15]. *R* is the ideal gas constant, *T* is the temperature, *a*_i_ is the activity of metabolite *i* (represented by the metabolite’s concentration), and $s_{i}$ is the stoichiometric coefficient for metabolite *i* in a certain reaction. For all reactions, the reactant and product concentrations were set to 0.1 M and 10^−6^ M, respectively. These two values are considered to be the upper and lower bounds of the concentration of metabolites under physiological conditions. The water activity was set to 1 M, which is an essential assumption of eQuilibrator [24,25]. Reactions with positive free energy were removed from the background metabolism pool. About 40% of the biosphere-level metabolic network reactions had no accurate free energy estimation (3122/7376). The background metabolism pools including and excluding these reactions were both analyzed in this study. The free energy data of the reactions can be found in Table S2.

It should be noted that the change in Gibbs free energy (ΔG) contains two components: entropy change (ΔS) and enthalpy change (ΔH). However, data of ΔS are not available for most reactions so it was not taken into account when using eQuilibrator to calculate ΔG in different temperatures [24,25]. This simplification may cause some reactions to be incorrectly defined as thermodynamically favored.

## 3. Results

All chemical reactions are constrained by the thermodynamic rules. A reaction with a positive standard change in Gibbs free energy (ΔG > 0) cannot proceed spontaneously. In modern organisms, these uphill reactions are usually coupled with exergonic reactions such as ATP hydrolysis to become energetically favorable. However, such stably coupled reaction systems may not have been available on primitive earth [28]. Therefore, the thermodynamic constraints could be an important limiting factor for primitive metabolism [13,14,15].

**Figure 1:**
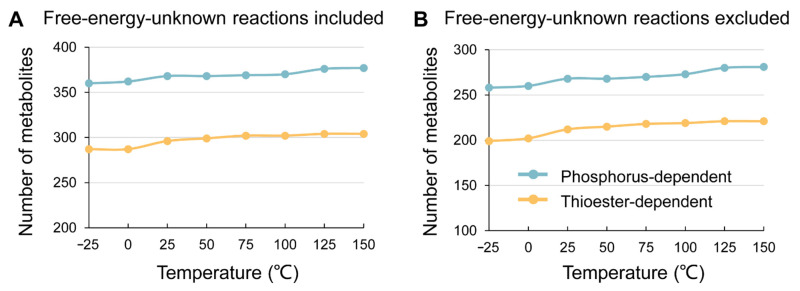
The scale of thermodynamically constrained networks at different temperatures. The figure shows the number of metabolites of the networks at different temperatures. (**A**) Including the reactions without accurate free energy estimation; (**B**) excluding the reactions without accurate free energy estimation. The green lines represent the phosphate-dependent networks, while the yellow lines represent the thioester-dependent networks. With the increase in temperature, the scales of the networks increase slightly.

Existing organisms were found to survive from −25 °C to over 120 °C. *Planococcus halocryophilus* Or1, a bacterium isolated from the salty water veins of high Arctic permafrost, can remain metabolically active at −25 °C [29]. Hyperthermophilic archaea *Methanopyrus kandleri* strain 116 can proliferate at 122 °C [30]. Therefore, we simulated the expansion of the phosphate-dependent and thioester-dependent networks at the temperatures from −25 °C to 150 °C. Using Goldford et al.’s method [14], we calculated the Δ*G* of the metabolic reactions at −25 °C, 0 °C, 25 °C, 50 °C, 75 °C, 100 °C, 125 °C, and 150 °C, respectively. As shown in Figure 1, from −25 °C to 150 °C, the scales of both phosphorus- and thioester-dependent networks only exhibit slight increases, regardless of whether the reactions include accurate free energy estimation or not.

**Figure 2:**
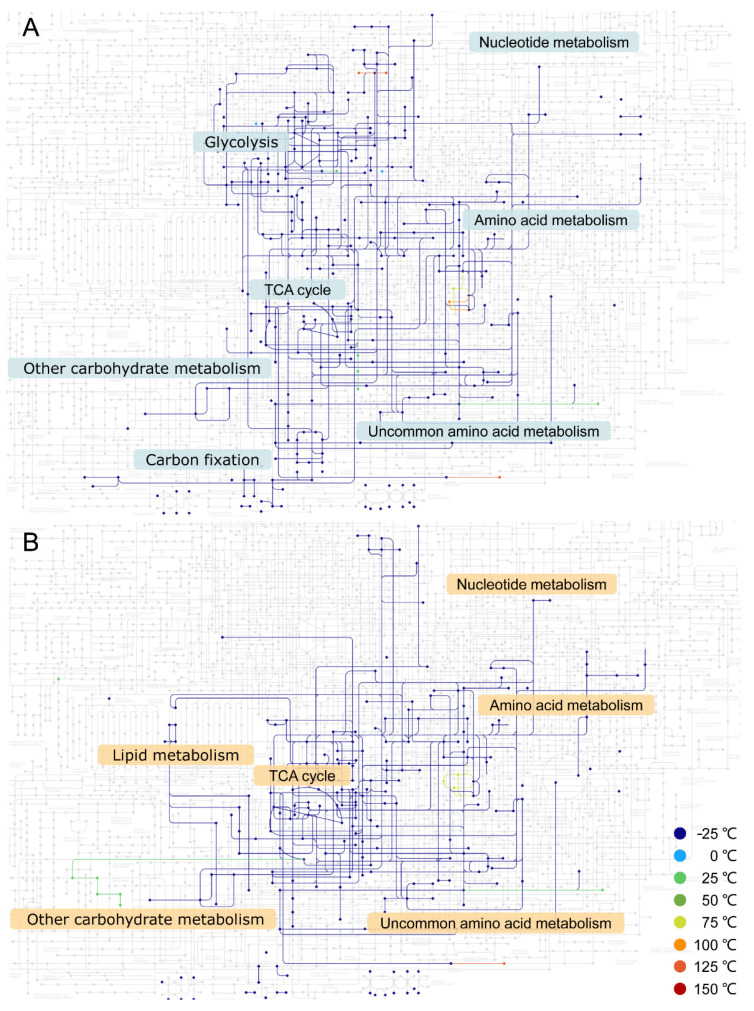
Metabolic networks at different temperatures. (**A**) Phosphate-dependent network; (**B**) thioester-dependent network. Reactions without accurate free energy estimation are included. The metabolites and reactions that appeared at different temperatures are represented by dots and lines of different colors, respectively. Light gray represents KEGG reference metabolites and reactions unavailable during the network expansions. Most reactions and metabolites can be achieved at −25 °C. One metabolite can be displayed as multiple nodes in the figure. The reactions and metabolites in different networks can be found in Table S3.

When including the reactions without accurate free energy estimation, the scale of the phosphate-dependent network obtained at −25 °C is close to the thermodynamically constrained network constructed in our previous study (360 vs. 338 metabolites) [15]. At this temperature, this network is mainly composed of reactions that participate in glycolysis, the tricarboxylic acid (TCA) cycle, and carbon fixation, which supply the basic carbohydrates and energy to living systems. They can also produce eight proteinogenic amino acids (Figure 2A, Table S3). The reactions that are only thermodynamically feasible at temperatures higher than −25 °C provide 17 new metabolites (Table S3). Seven of these metabolites are involved in the amino acid metabolism, but none of them are proteinogenic amino acids. The other metabolites generated at higher temperatures are scattered across different pathways. When excluding the reactions without accurate free energy estimation, the network keeps the functions mentioned above, and the temperature increase has no significant influence on these functions (Figure S1A, Table S3). The thioester-dependent networks can produce two more proteinogenic amino acids but lack glycolysis-related carbohydrates. These functions are also not greatly influenced by the change in temperature (Figure 2B and Figure S1B, and Table S3).

Nucleotides are the basic components of RNA and constitute the cofactors of many proteins, so they are of great significance in the origin of life [31]. As there is no phosphorus in the thioester-dependent networks, it is obviously impossible for them to synthesize nucleotides (Figure 2B and Figure S1B). In fact, they cannot even synthesize ribose (Table S3). The phosphate-dependent networks can generate ribose 5-phosphate (KEGG ID C00117) at all tested temperatures, which is a precursor required for the synthesis of nucleotides (Table S3). Moreover, ribose 5-phosphate can be further phosphorylated to 5-phosphoribosyl diphosphate (PRPP) through KEGG reaction R01049, which is necessary for both purine and pyrimidine synthesis (Figure S2). Under a physiological condition, ATP provides the phosphate group and energy for the reaction. ATP is located at the end of one branch of the KEGG nucleotide synthesis pathway. Therefore, ATP cannot be used in a reaction that is located at the upstream end of the pathway, which prevented the phosphate-dependent networks from expanding along this path. However, there may be other ways to achieve the phosphorylation of ribose 5-phosphate. In fact, glucose-6-phosphate hydrolysis can provide phosphate and energy. We constructed such a reaction: ribose 5-phosphate + 2 glucose 6-phosphate => PRPP + 2 glucose. Under the substance concentration condition used in the network expansion simulation, this reaction is thermodynamically favorable even at −25 °C (with a free energy of −33.56 kJ/mol), implying that glucose-6-phosphate may play a role like ATP and facilitate the phosphate-dependent network to generate the nucleotides.

In modern life, ATP plays multiple roles in metabolism. In the background metabolism pool used in this study, ATP is the reactant or product of 471 reactions. These reactions cannot be reached by the network expansion because ATP is not available in the simulation. In primitive metabolism, other phosphorus-containing compounds with simpler structures may be more prevalent than ATP, and played similar roles to it in these reactions [32]. As shown in the fictional reaction to synthesize PRPP, their products may be obtained through reactions that do not rely on ATP. However, the possibility that ATP existed in the prebiotic world cannot be completely ruled out, because the abiotic synthesis of ATP from simple inorganic substances may be achieved in the prebiotic environment [33,34,35]. Therefore, we replaced the glucose-6-phosphate in the “seeds” with ATP and performed network expansions. The obtained metabolic networks have about 200 more metabolites than the phosphorus-dependent networks at the same temperatures. For these ATP-dependent networks, from −25 °C to 150 °C, only 24 metabolites were added (including the reactions without accurate free energy estimation, Table S3), showing a limited influence of temperature.

These findings showed that, at least in terms of thermodynamics, the scale and main functions of the simulated metabolic networks are slightly affected by temperature. The free energy characteristics (positive or negative) of most reactions do not change within the examined temperature range (Table S2), so the feasibility of these reactions is not largely influenced by temperature. These results suggest that, whether it is dependent on phosphate or thioester, metabolism may have originated in a relatively wide temperature range.

## 4. Discussion

There are many different opinions on the temperature at the origin of life. Darwin speculated that the first living organisms evolved in “warm little ponds” [36]. The Miller–Urey experiment showed that a warm, lightning-filled atmosphere that may have existed on primitive Earth can produce important biomolecules [37]. Hydrothermal environments were also “hot” in the area of the origin of life. Terrestrial hydrothermal fields and submarine hydrothermal vents can provide materials, energy, reducing power, and pH gradients, which may facilitate the synthesis of basic biomolecules [38,39,40]. Moreover, terrestrial hydrothermal fields can evaporate water to become dry and get wet by rain, which formed a natural dry–wet cycle [41]. Dry–wet cycles are very important for the synthesis of nucleotides under metal catalysis [42]. These effects are also significant for the polymerization of biological macromolecules such as peptides [43,44] and polynucleotides [41,45].

A criticism of the hydrothermal origin of life is that although the structures of most small molecule metabolites are stable, the high temperature can accelerate their reactions with other substances, such as water, and thus causes their thermal instability [46]. Miller and colleagues argued that important metabolites such as ribose and ATP are thermally unstable, thus life was not likely to originate at high temperatures [47,48]. However, the meaning of “stable” is relative. Some metabolites in metabolic pools have very short turnover times. Metabolites that can exist longer than their turnover time in organisms should have a chance to participate in metabolic reactions as reactants. Based on this principle, Bains et al. predicted the degradation rates of 63 metabolites at different temperatures and compared them with the intracellular half-life of these metabolites [46]. As a result, most of the metabolites were found to be unable to exist longer than their intracellular half-life at temperatures above 150–180 °C. Therefore, Bains et al. suggested that this temperature range is the upper limit of biochemistry.

Some basic materials for life also can be generated at cold temperatures. HCN polymerization may be an important procedure in biomolecule synthesis [49]. However, this process not likely to occur in warm environments due to the fast hydrolysis of HCN [50]. It has been found that the eutectic freezing of HCN and water at –21 °C can concentrate the former and promote its polymerization [50]. Moreover, eutectic freezing of NH_4_CN solutions can generate nucleic acid bases and amino acids [51,52].

In addition to the conditions required for the synthesis of biomolecules, the living environment of modern organisms can also be used as a clue to explore the temperature for the origin of life. Previously, thermophilic bacteria and archaea were considered to be located near the roots of the phylogenetic tree, suggesting that life originated in hot temperatures [1,2,3]. However, in a newer rRNA-based phylogenetic tree of bacteria, mesophilic species rather than thermophiles are the closest branches to the root, showing that the thermophiles are latecomers in evolution [4]. Several studies inferred the temperature of the habitat for the LUCA based on the deduced rRNA GC contents or protein amino acid compositions, but no agreement has been reached [5,6,7].

The inconsistent results from gene sequence-based studies can be attributed to the uncertain early life evolution trajectory, which is difficult to properly characterize or experimentally test [53]. Compared with the genetic system, metabolic reactions may have an earlier origin and are more common among different species. Therefore, metabolism could be a window to detect the properties of the earliest life. In this study, the influence of temperature on metabolism was analyzed in terms of thermodynamics. We found that whether glucose 6-phosphate or thioester was used to alleviate the thermodynamic bottleneck of network expansion, there is no significant difference in the function and scale of the networks generated at different temperatures. These results imply that the origin of metabolism could have occurred in a relatively wide temperature range.

Although the thermodynamic data of most biochemical reactions at different temperatures are very scarce, several reactions that are crucial for network expansion may have this kind of information. To identify these network scale-limiting reactions, the network expansion was re-performed several times, wherein the reactions from the background metabolism pool were removed one by one. We found that the deletion of most reactions did not cause a significant reduction in the generated network, except for a few (Table S4). When excluding the reactions without accurate free energy estimation, reactions R00874, R01519, R01538, and R08570 are necessary for maintaining the scale of the phosphate-dependent network. Without any of these reactions, the network expansion ceased with up to eighty-nine metabolites. These four reactions are related to glucose metabolism. In R00874, glucose reacts with fructose to form gluconolactone and glucitol. The other three reactions are components of the pentose phosphate pathway, which starts with glucose and generates multiple important metabolites. For the thioester-dependent networks, reaction R00212 is necessary for network expansion. Without this reaction, the produced networks contained only dozens of metabolites. In this reaction, acetyl-CoA reacts with formate and generates CoA and pyruvate. These results showed that certain reactions can indeed deeply influence the expansion of metabolic networks. We tried to find out more about their feasibility at different temperatures, but no useful information was found. Further study on the thermodynamics of these key reactions may provide a better understanding of the temperature of the environment where life originated.

Thermodynamics is a very fundamental property for chemical reactions. Therefore, this study may also be meaningful for finding exoplanet life. Based on planetary physical parameters such as temperature, Lingam and Loeb formulated likelihood functions estimating the possibility of the existence of life on a certain planet [54]. In the calculation, they referred to the temperature range of the living creatures on Earth (262 to 395 K). Temperatures beyond this range will lead to a rapid decline in the possibility of life. The tested temperature range in this study is a bit wider (−25 to 150 °C, i.e., 248.15 to 423.15 K). The present analysis suggests that metabolic networks may originate in this range. In our simulation, the expansion of metabolic networks does not reject low temperature, which increases the possibility of life on cold celestial bodies. Primitive metabolism may occur in the saltwater lakes on Mars, the liquid methane of Titan, or a liquid ammonia ocean elsewhere.

In addition to the above discussion, the interplays between temperature and other factors such as the phase change of water (e.g., dry–wet cycle and eutectic freezing, as discussed above) and redox potential can promote metabolic reactions. In Goldford et al.’s study, redox potential had a decisive influence on the scale of the metabolism networks [14]. Temperature gradients can cause electrochemical potentials which can be used as a thermodynamic driving force. For example, the high temperature, pressure, and pH of deep sea alkaline hydrothermal vents elevate the redox potential of H_2_ oxidation, which can be used as an electrochemical driving force for the abiotic reduction of CO_2_ [55,56]. Intriguingly, temperature may also shape the structure of peptides [57], which could serve as catalysts in prebiotic metabolism. To date, most studies on the origin of metabolism have not considered the interactions between temperature and other environmental factors. Goldford et al.’s study investigated several environmental factors, but they are still independent of each other. The interplays between temperature and other factors and how they affect metabolism deserve more in-depth studies. Moreover, the feasibility of chemical reactions is also dependent on their kinetics. Thermodynamically favored reactions may have high energy barriers between the reactants and products, which make the reactions unfeasible in terms of kinetics. In future studies, we will explore the influence of temperature on the kinetics of key metabolic reactions by calculating the activation energies for the reactions.

## Supplementary Materials

The following are available online at https://www.mdpi.com/article/10.3390/life11080738/s1, Figure S1: Metabolic networks at different temperatures. Figure S2: The link between phosphate-dependent networks and nucleotide metabolic pathways. Table S1: Background metabolism pool. Table S2: Gibbs free energy of the reactions at different temperatures. Table S3: Metabolites and reactions at different temperatures.

## References

[1] Woese C.R. (1987). Bacterial evolution. Microbiol. Rev..

[2] Achenbach-Richter L., Gupta R., Zillig W., Woese C.R. (1988). Rooting the archaebacterial tree: The pivotal role of *Thermococcus celer* in archaebacterial evolution. Syst. Appl. Microbiol..

[3] Pace N.R. (1991). Origin of life—Facing up to the physical setting. Cell.

[4] Brochier C., Philippe H. (2002). Phylogeny: A non-hyperthermophilic ancestor for bacteria. Nature.

[5] Galtier N., Tourasse N., Gouy M. (1999). A nonhyperthermophilic common ancestor to extant life forms. Science.

[6] Boussau B., Blanquart S., Necsulea A., Lartillot N., Gouy M. (2008). Parallel adaptations to high temperatures in the Archaean eon. Nature.

[7] Groussin M., Boussau B., Charles S., Blanquart S., Gouy M. (2013). The molecular signal for the adaptation to cold temperature during early life on Earth. Biol. Lett..

[8] Eck R.V., Dayhoff M.O. (1966). Evolution of the structure of ferredoxin based on living relics of primitive amino acid sequences. Science.

[9] Hartman H. (1975). Speculations on the origin and evolution of metabolism. J. Mol. Evol..

[10] Morowitz H.J., Kostelnik J.D., Yang J., Cody G.D. (2000). The origin of intermediary metabolism. Proc. Natl. Acad. Sci. USA.

[11] Lanier K.A., Williams L.D. (2017). The origin of life: Models and data. J. Mol. Evol..

[12] Ebenhöh O., Handorf T., Heinrich R. (2004). Structural analysis of expanding metabolism networks. Genome Inform..

[13] Goldford J.E., Hartman H., Smith T.F., Segrè D. (2017). Remnants of an ancient metabolism without phosphate. Cell.

[14] Goldford J.E., Hartman H., Marsland R., Segrè D. (2019). Environmental boundary conditions for the origin of life converge to an organo-sulfur metabolism. Nat. Ecol. Evol..

[15] Tian T., Chu X.-Y., Yang Y., Zhang X., Liu Y.-M., Gao J., Ma B.-G., Zhang H.-Y. (2019). Phosphates as energy sources to expand metabolism networks. Life.

[16] Schwartz A.W. (2006). Phosphorus in prebiotic chemistry. Philos. Trans. R. Soc. B.

[17] De Duve C. (2003). A research proposal on the origin of life. Orig. Life Evol. Biosph..

[18] Piast R.W., Wieczorek R.M. (2017). Origin of life and the phosphate transfer catalyst. Astrobiology.

[19] Glindemann D., De Graaf R.M., Schwartz A.W. (1999). Chemical reduction of phosphate on the primitive earth. Orig. Life Evol. Biosph..

[20] Pasek M.A. (2017). Schreibersite on the early Earth: Scenarios for prebiotic phosphorylation. Geosci. Front..

[21] Yamagata Y., Watanabe H., Saitoh M., Namba T. (1991). Volcanic production of polyphosphates and its relevance to prebiotic evolution. Nature.

[22] Benner S.A., Kim H.J., Carrigan M.A. (2012). Asphalt, water, and the prebiotic synthesis of ribose, ribonucleosides, and RNA. Acc. Chem. Res..

[23] Ogata H., Goto S., Sato K., Fujibuchi W., Bono H., Kanehisa M. (1999). KEGG: Kyoto Encyclopedia of Genes and Genomes. Nucleic Acids Res..

[24] Flamholz A., Noor E., Bar-Even A., Milo R. (2012). eQuilibrator—The biochemical thermodynamics calculator. Nucleic Acids Res..

[25] Noor E., Haraldsdóttir H.S., Milo R., Fleming R.M.T. (2013). Consistent estimation of Gibbs energy using component contributions. PLoS Comput. Biol..

[26] Coggins A.J., Powner M.W. (2017). Prebiotic synthesis of phosphoenol pyruvate by phosphorylation controlled triose glycolysis. Nat. Chem..

[27] Keller M.A., Turchyn A.V., Ralser M. (2014). Non-enzymatic glycolysis and pentose phosphate pathway-like reactions in a plausible Archean ocean. Mol. Syst. Biol..

[28] Bar-Even A., Flamholz A., Noor E., Milo R. (2012). Thermodynamic constraints shape the structure of carbon fixation pathways. BBA Bioenergetics.

[29] Mykytczuk N.C.S., Foote S.J., Omelon C.R., Southam G., Greer C.W., Whyte L.G. (2013). Bacterial growth at −15 °C; molecular insights from the permafrost bacterium *Planococcus halocryophilus* Or1. ISME J..

[30] Takai K., Nakamura K., Toki T., Tsunogai U., Miyazaki M., Miyazaki J., Hirayama H., Nakagawa S., Nunoura T., Horikoshi K. (2008). Cell proliferation at 122 °C and isotopically heavy CH_4_ production by a hyperthermophilic methanogen under high-pressure cultivation. Proc. Natl. Acad. Sci. USA.

[31] Chu X.Y., Zhang H.Y. (2020). Cofactors as molecular fossils to trace the origin and evolution of proteins. ChemBioChem.

[32] Whicher A., Camprubi E., Pinna S., Herschy B., Lane N. (2018). Acetyl phosphate as a primordial energy currency at the origin of life. Orig. Life Evol. Biosph..

[33] Yamagata Y. (1999). Prebiotic formation of ADP and ATP from AMP, calcium phosphates and cyanate in aqueous solution. Orig. Life Evol. Biosph..

[34] Akouche M., Jaber M., Maurel M.C., Lambert J.F., Georgelin T. (2017). Phosphoribosyl pyrophosphate: A molecular vestige of the origin of life on minerals. Angew. Chem. Int. Ed. Engl..

[35] Yadav M., Kumar R., Krishnamurthy R. (2020). Chemistry of abiotic nucleotide synthesis. Chem. Rev..

[36] Peretó J., Bada J.L., Lazcano A. (2009). Charles Darwin and the origin of life. Orig. Life Evol. Biosph..

[37] Miller S.L. (1953). A production of amino acids under possible primitive earth conditions. Science.

[38] Deamer D., Damer B., Kompanichenko V. (2019). Hydrothermal chemistry and the origin of cellular life. Astrobiology.

[39] Damer B., Deamer D. (2020). The hot spring hypothesis for an origin of life. Astrobiology.

[40] Omran A., Pasek M. (2020). A constructive way to think about different hydrothermal environments for the origins of life. Life.

[41] Ross D., Deamer D. (2016). Dry/wet cycling and the thermodynamics and kinetics of prebiotic polymer synthesis. Life.

[42] Cheng C., Fan C., Wan R., Tong C., Miao Z., Zhao Y. (2002). Phosphorylation of adenosine with trimetaphosphate under simulated prebiotic conditions. Orig. Life. Evol. Biosph..

[43] Fox S., Harada K., Kendrick J. (1959). Production of spherules from proteinoids and hot water. Science.

[44] Yu S.-S., Solano M., Blanchard M.K., Soper-Hopper M., Krishnamurthy R., Fernandez F.M., Hud N.V., Schork F.J., Grover M.A. (2017). Elongation of model prebiotic proto-peptides by continuous monomer feeding. Macromolecules.

[45] De Guzman V., Vercoutere W., Shenasa H., Deamer D.W. (2014). Generation of oligonucleotides under hydrothermal conditions by non-enzymatic polymerization. J. Mol. Evol..

[46] Bains W., Xiao Y., Yu C. (2015). Prediction of the maximum temperature for life based on the stability of metabolites to decomposition in water. Life.

[47] Miller S.L., Bada J.L. (1988). Submarine hot springs and the origin of life. Nature.

[48] Miller S.L., Lazcano A. (1995). The origin of life—Did it occur at high temperatures?. J. Mol. Evol..

[49] Matthews C.N., Minard R.D. (2006). Hydrogen cyanide polymers, comets and the origin of life. Faraday Discuss..

[50] Miyakawa S., Cleaves H.J., Miller S.L. (2002). The cold origin of life: A. implications based on pyrimidines and purines produced from frozen ammonium cyanide solutions. Orig. Life Evol. Biosph..

[51] Miyakawa S., Cleaves H.J., Miller S.L. (2002). The cold origin of life: B. implications based on pyrimidines and purines produced from frozen ammonium cyanide solutions. Orig. Life Evol. Biosph..

[52] Levy M., Miller S.L., Oró J. (1999). Production of guanine from NH_4_CN polymerizations. J. Mol. Evol..

[53] Doolittle W.F. (1999). Phylogenetic classification and the universal tree. Science.

[54] Lingam M., Loeb A. (2018). Physical constraints on the likelihood of life on exoplanets. Int. J. Astrobiol..

[55] Ooka H., McGlynn S.E., Nakamura R. (2019). Electrochemistry at deep-sea hydrothermal vents: Utilization of the thermodynamic driving force towards the autotrophic origin of life. Chem. Electro. Chem..

[56] Boyd E.S., Amenabar M.J., Poudel S., Templeton A.S. (2020). Bioenergetic constraints on the origin of autotrophic metabolism. Philos. Trans. A Math. Phys. Eng. Sci..

[57] Brack A., Spach G. (1981). Multiconformational synthetic polypeptides. J. Amer. Chem. Soc..

