# Supplementary material for: Plausibility of Early Life in a Relatively Wide Temperature Range: Clues from Simulated Metabolic Network Expansion
Source: Life (Basel). 2021 Jul 24;11(8):738. doi: 10.3390/life11080738 (PMC8398716; doi:10.3390/life11080738)
Supplement: Supplementary file 1 [file life-11-00738-s001.zip › Supplementary Figures.pdf]

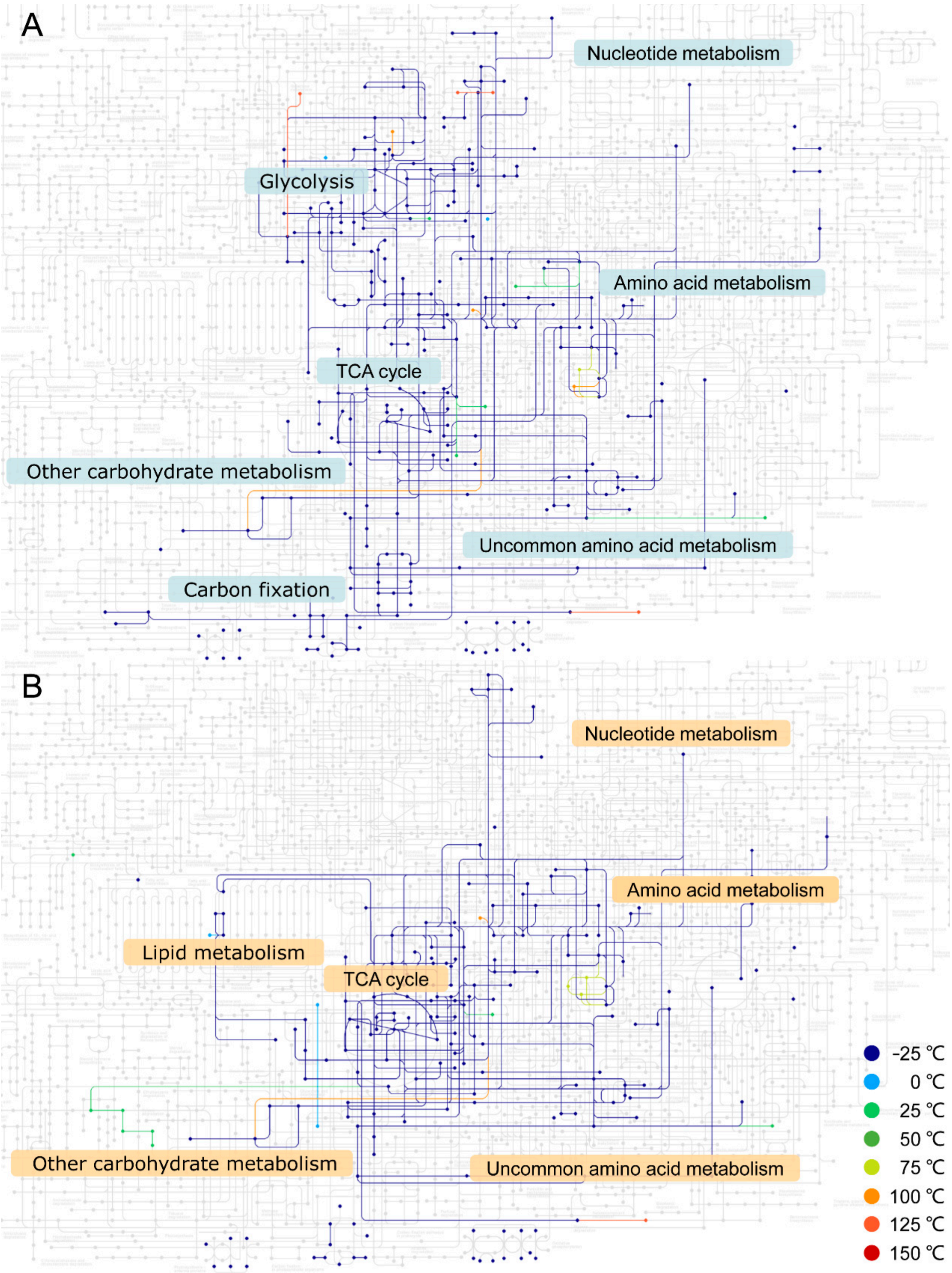

Figure S1. Metabolic networks at different temperatures. A) phosphate-dependent network. B) thioester-dependent network. Reactions without accurate free energy estimation are excluded. The metabolites and reactions that appeared at different temperatures are represented by dots and lines of different colors correspondently. One metabolite can be displayed as multiple nodes in the figure.

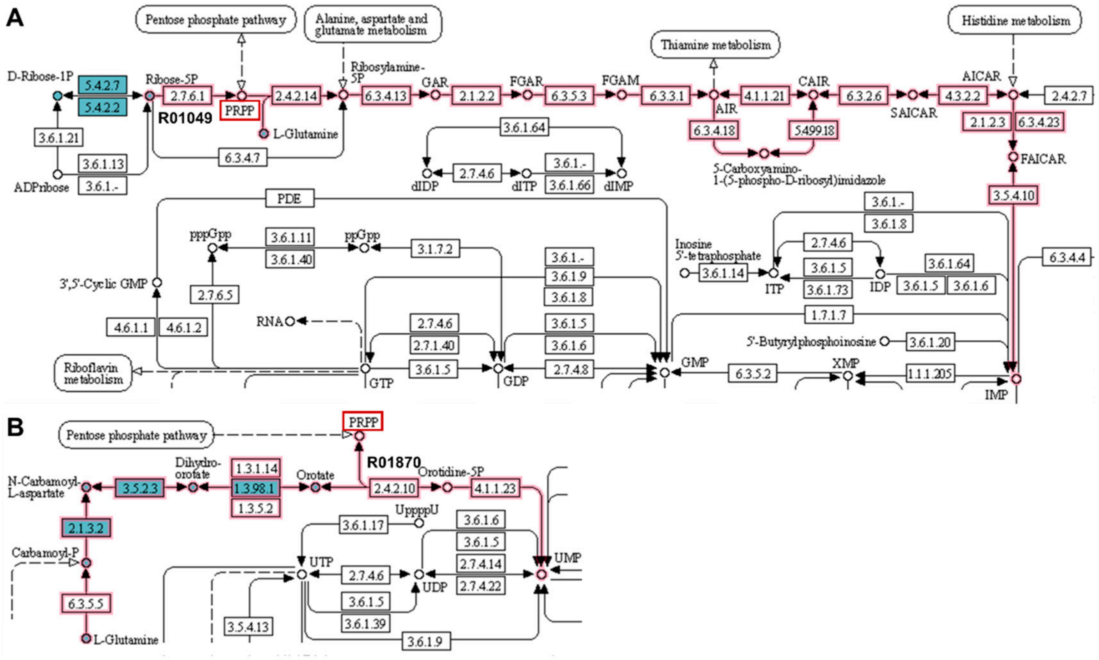

Figure S2. The link between phosphate-dependent network and nucleotide metabolic pathways. A) and B) show the relation of the phosphate-dependent network with purine and pyrimidine metabolic pathways, respectively. Metabolites and reactions belong to the phosphate-dependent network are marked in green. The pink-shaded routes lead to the first purine (IMP) and the first pyrimidine (UMP) in biosynthesis. R01049 and R01870 are the reactions that hinder the expansion of the network to nucleotide metabolism. The limiting metabolite 5-phosphoribosyl diphosphate (PRPP) is marked with red boxes.
